# Time-Dependent Compensatory Responses to Chronic Neuroinflammation in Hippocampus and Brainstem: The Potential Role of Glutamate Neurotransmission

**DOI:** 10.4172/2161-0460.1000110

**Published:** 2013-03-28

**Authors:** Holly M. Brothers, Isabelle Bardou, Sarah C. Hopp, Yannick Marchalant, Roxanne M. Kaercher, Sarah M. Turner, Mollie R. Mitchem, Kristina Kigerl, Gary L. Wenk

**Affiliations:** 1 Department of Psychology, Ohio State University, Columbus, OH, USA; 2 NICN, UMR 6184, Université de la Méditerranée, Marseille, France; 3 Department of Neuroscience, Ohio State University, Columbus, OH, USA

**Keywords:** Neuroinflammation, Rat, Lipopolysaccharide, Alzheimer, Parkinson, Glutamate, Excitatory amino acid transporter, SNAP25

## Abstract

Chronic neuroinflammation is characteristic of neurodegenerative diseases and is present during very early stages, yet significant pathology and behavioral deficits do not manifest until advanced age. We investigated the consequences of experimentally-induced chronic neuroinflammation within the hippocampus and brainstem of young (4 mo) F-344 rats. Lipopolysaccharide (LPS) was infused continuously into the IV^th^ ventricle for 2, 4 or 8 weeks. The number of MHC II immunoreactive microglia in the brain continued to increase throughout the infusion period. In contrast, performance in the Morris water maze was impaired after 4 weeks but recovered by 8 weeks. Likewise, a transient loss of tyrosine hydroxylase immunoreactivity in the substantia nigra and locus coeruleus was observed after 2 weeks, but returned to control levels by 4 weeks of continuous LPS infusion. These data suggest that direct activation of microglia is sufficient to drive, but not sustain, spatial memory impairment and a decrease in tyrosine hydroxylase production in young rats. Our previous studies suggest that chronic neuroinflammation elevates extracellular glutamate and that this elevation underlies the spatial memory impairment. In the current study, increased levels of GLT1 and SNAP25 in the hippocampus corresponded with the resolution of performance deficit. Increased expression of SNAP25 is consistent with reduced glutamate release from axonal terminals while increased GLT1 is consistent with enhanced clearance of extracellular glutamate. These data demonstrate the capacity of the brain to compensate for the presence of chronic neuroinflammation, despite continued activation of microglia, through changes in the regulation of the glutamatergic system.

## Introduction

Chronic neuroinflammation is now widely considered a risk factor for many age-associated neurodegenerative diseases, including Alzheimer’s disease (AD) and Parkinson’s disease (PD) [1–4]. Aberrant extracellular proteins such as β-amyloid and α-synuclein activate resident microglia and initiate neuroinflammation in AD and PD, respectively [5,6]. Increased levels of pro-inflammatory factors have been found in cerebral spinal fluid and post-mortem brain tissue of AD and PD patients [7–9]. Activated microglia are detectable many years prior to the onset of characteristic neuropathological and symptomatic changes diagnostic for AD and PD and are found in brain regions that ultimately show significant neuropathology, the hippocampus and substantia nigra (SN), respectively, as demonstrated by positron emission tomography (PET) studies [6,10–16]. These vulnerable brain regions are likely exposed to a pro-inflammatory environment for many decades [17–19] yet do not show significant pathology until advanced age [12,15,20]. The young adult brain appears to recover from exposure to pro-inflammatory stimuli.

We have produced a pro-inflammatory environment in young male rats by infusion of lipopolysaccharide (LPS) into the IV^th^ ventricle, and have systematically documented a slowly evolving and region-specific series of changes induced by the chronic infusion of picomolar levels of LPS; these changes represent a complex interplay of glial and neuronal interactions leading to a series of compensatory changes that transpire in the presence of continued stimulation of microglia. After only two days of LPS infusion, activated microglia were seen diffusely scattered throughout the brain, suggesting that the LPS had spread throughout both cerebral hemispheres within a relatively short period of time [21]. After 4 weeks of LPS infusion, the number of activated microglia gradually decreased in all regions with some key exceptions: the greatest inflammatory response was concentrated within the hippocampus [21]. The increased number of activated microglia correlated with a reduction in the number of N-methyl-d-aspartate (NMDA) glutamate receptors within the dentate gyrus (DG) and CA3 hippocampal areas without evidence of neuronal loss [22], an impairment in spatial memory but not object recognition performance [23], impaired long-term potentiation [24], the loss of layer II and III pyramidal cells in entorhinal cortex [24] and elevated levels of mRNA for multiple pro-inflammatory cytokines [23]. After four weeks of LPS infusion, MRI studies identified enlarged lateral ventricles with shrinkage of the temporal lobe regions [25]. Chronic infusion of LPS into the basal forebrain produced a selective decline in the number of neurons displaying choline acetyltransferase-immunoreactivity; the loss of choline acetyltransferase-immunoreactive cells was time-dependent but not dose-dependent [21,26]. These effects of LPS were reproduced by chronic infusion of tumor necrosis factor-α (TNFα), which produced identical changes in the number of choline acetyltransferase-immunoreactive neurons within the basal forebrain [27]. Taken together, these findings suggest that LPS quickly initiates a cascade of biochemical processes that show time-dependent, region-specific and cell selective changes that progress during four weeks of LPS infusion [21,23]. The current study extended the duration of continuous LPS exposure to 8 weeks and investigated behavioral, cellular and molecular consequences of neuroinflammation in the hippocampus and brainstem, regions that show a peculiar vulnerability to age-associated neurodegeneration [7,8].

## Methods

### Subjects and surgical procedures

110 young (4 mo) male F-344 rats (Harlan, Indianapolis., IN) survived throughout the entire surgical implantation, infusion and behavioral testing periods. Each rat was singly housed in Plexiglas cages with ad libitum access to food and water. The rats were maintained on a 12/12-h light–dark cycle with lights off at 09:00 in a temperature-controlled room (22°C). All rats were given health checks, monitored and periodically handled throughout the study. All experimental protocols described here were approved by the Institutional Animal Care and Use Committee of The Ohio State University.

Rats were allowed one week to acclimate to their new environment before surgery. Each rat was anesthetized using isoflurane gas (2.5% metofane by volume in 2 liters/min compressed oxygen) and a cannula was surgically implanted into the IV^th^ ventricle (−2.5 AP, −7 DV relative to lambda) and attached to an osmotic minipump (model #2006, Alzet, Durect Corp, Cupertino, CA) as previously described. The mean fill volume for the #2006 minipump is 243 μl and flow rate is 0.15 μl/hr; thus continuous infusion could last at least nine weeks; therefore, all rats were sacrificed prior to the cessation of LPS infusion. Artificial cerebrospinal fluid (aCSF, 140 mM NaCl, 3.0 mM KCl, 2.5 mM CaCl_2_, 1.0 mM MgCl_2_ and 1.2 mM Na_2_HPO_4_ adjusted to pH 7.4) or LPS (0.25μg/hr, dissolved $1.\bar{66}\;\text{mg}/\text{ml}$ into aCSF; *E. coli* serotype 055:B5, TCA extraction, Sigma, St. Louis, MO) were chronically infused for either 2, 4 or 8 weeks, creating 6 experimental groups: aCSF 2w (n=10), LPS 2w (n=16), aCSF 4w (n=21), LPS 4w (n=31), aCSF 8w (n=14) and LPS 8w (n=18). Post-operative care included recovery in a warm environment, local application of lidocaine 1% solution and topical anesthetic to the incision sites and sub-cutaneous injection of 2 ml isotonic saline to prevent dehydration. Body weights were monitored daily and nutritional supplements were provided as necessary during the infusion period.

### Behavioral Analysis

#### Morris water maze

Hippocampal-sensitive spatial memory was evaluated by performance in the Morris water maze. Four days of testing were conducted prior to sacrifice, 4 or 8 weeks after surgery according to Marchalant et al., [28]. Rats in the 2 week groups were not tested due to our concern that the effects of the surgery and LPS might interfere with performance. The pool (170 cm diameter with grey walls) was in the center of a room with multiple distal visual cues and a plastic board against the wall of the pool as a proximal visual cue. A hidden escape platform (10 cm diameter) was present in a constant location, submerged 2.5 cm below the water surface. The rats were tracked using Noldus Ethovision 3.1 tracking and analysis system (Noldus, Leesburg, VA). Initially, rats were placed on the hidden platform for 30 sec. Each rat then performed three training blocks per day of two trials per block (6 total trials per day) over 4 days that were initiated from one of seven locations varied randomly from trial to trial. After the rat found the escape platform or swam for a maximum of 60 sec, it was allowed to remain on the platform for 30 sec. A probe trial of 60 sec in which the escape platform was removed was conducted on the 4^th^ day after the completion of 6 trials. Two visible platform trials were then conducted in which the platform was raised 2 cm above the surface of the water, marked with a latex glove and moved randomly. Latency to find the hidden platform, swim speed, time spent in the perimeter of the pool (thigmotaxis) and time spent within the region of the removed platform during the probe trial were analyzed.

### Histology

Following completion of behavioral testing, rats were anesthetized with isoflurane and were transcardially perfused (10 ml/min) with cold saline (0.9%, 80 ml) containing heparin (1 U/ml), followed by paraformaldehyde (4% dissolved in 0.1M phosphate buffer, pH 7.4, 120 ml). Serial coronal sections of 40 μm were obtained using a vibratome (Leica) and stored at −20 °C in anti-freeze solution (0.5 M phosphate buffer with 30% glycol ethylene and 30% glycerol).

Histological analysis using single- and double-chromogenic staining as well as fluorescence staining was performed as previously described [29,30]. For chromogenic staining, free-floating tissues were rinsed in PBS (0.01 M), quenched for endogenous peroxidase activity (50% methanol, 0.3% triton and 0.3% H_2_O_2_), blocked with normal goat serum (5%), and incubated with primary antibody over one or two nights at 4°C. Thereafter, sections were incubated with biotinylated secondary antibody for 1.5 hr, rinsed, incubated for 1 hr with avidin-biotinylated horseradish peroxidase (ABC kit, Vector, Burlingame, CA), rinsed, and visualized by incubation with 0.05% 3,3-diaminobenzidine tetrahydrochloride (DAB, Vector, Burlingame, CA) as chromogen. Double-peroxidase stained sections were then placed into another primary antibody processed as described above and visualized with SG Blue (Vector, Burlingame, CA). After the final rinse, sections were mounted onto gel coated slides, rehydrated, counterstained with cresyl violet, dehydrated, coverslipped with cytoseal (Allan Scientific, Kalamazoo, MI) and examined using light microscopy. Fluorescent staining followed a similar initial procedure, however after the first rinse; tissues were mounted to gel-coated slides. Following incubation with primary antibody, tissues were incubated with avidin-biotin amplification system (Vector), incubated with secondary antibody then AB detection kit (Vector) and visualized using the TSA fluorescence system CY3 (PerkinElmer Life Sciences, Emeryville, CA) with the nuclei counterstain DAPI. No staining was detected in the absence of the primary or secondary antibodies. Confidence was established for antibodies that also produced bands at the expected kDa in Western blot.

Within the hippocampus, activated microglia cells were visualized with a mouse antibody directed against Class II major histocompatibility complex (MHC II, 1:400, Pharmingen, San Diego, CA). Dopamine-β-hydroxylase (DBH), an enzyme that produces norepinephrine from dopamine, was evaluated as a reporter of projection strength of LC neurons (anti-DBH, 1:2000, rabbit polyclonal, AbCam). Synaptosome associated protein 25 (SNAP25) is involved in vesicle docking, and was evaluated using rabbit anti-SNAP25 (1:750, AbCam, Cambridge, MA). The glutamate transporter GLT1 (excitatory amino acid transporter 2, EAAT2) was evaluated with guinea-pig anti-GLT1 (1:2000, Millipore). MHC II-immunoreactive (MHC II-IR) microglia were manually counted using MetaMorph imaging software (Universal Image Corp., West Chester, PA) in two representative hippocampal slices. SNAP25 and GLT1 were evaluated in two representative hippocampal slices per animal, with all tissues stained simultaneously and subjected to identical staining conditions, and then evaluated as staining density per area using Image J software (NIH) or Nikon Elements (Nikon, Melville, NY). We later stained for MHC II, SNAP25 and GLT1 in tissue from aged rats with identical procedure and incubation times, and included tissues from young rats from each group previously stained in order to confirm the conformity of the staining.

Within the SN, dopaminergic neurons were stained with rabbit anti-tyrosine hydroxylase (TH, 1:750, Millipore), a dopamine precursor, as well as MHC II and a stereological analysis was conducted on a group of tissues composed of 5 slices of 40μm separated by 200μm (every 6^th^ slice) across the SNpc using the optical fractionator technique (stereology module of an MCID 6.0 Elite; Imaging Research, St. Catharines, Ontario, Canada) according to [31,32]. Briefly, a counting frame of approximately 125,000 μm^3^ was applied and sampled 30% of the region of interest under 63X magnification on a light microscope (Zeiss) equipped with a motorized Z-axis and X-Y stage (Ludl Electronics, Hawthorne, NY). TH-IR neurons were included if the nucleus was in focus within the sampling volume and did not intersect the top or left boundaries of the counting frame, MHC II-IR microglia were included if the cell body met these criteria. Estimates of the total number of TH-IR neurons and MHC II-IR microglia per section where calculated using the equation: N=Q×1/hsf×1/asf×1/ssf, where N is the total estimated number of cells, Q equals the total number of cells sampled across 5 sections and hsf, asf, and ssf represent the tissue height, area, and section sampling fractions, respectively. In addition, phosphorylated TH (pTH), the active form of TH, was visualized using rabbit anti-pTH (1:500, Millipore) and evaluated as staining density per area.

### Cell culture

Nitric oxide (NO) release from BV-2 microglia cell culture: In order to confirm that LPS contained in the osmotic minipumps was still viable after a period of 2 to 8 weeks at body temperature, we filled osmotic mini-pumps with LPS and incubated them in a 0.9% saline solution at 37° C for 4, 6 or 8 weeks. Freshly prepared LPS (100 ng/ml) and samples of LPS from each mini-pump were investigated for their ability to induce the release of nitric oxide (NO) by microglia in a BV-2 culture. Microglia cells were plated 100,000 cells per well in a 96-well plate and incubated with media (DMEM, 10% FBS, 1% PenStep and 1% Glutamax) or 200 μl of LPS sample for 24 hrs. NO release due to LPS exposure was examined using the Greiss Assay kit (Invitrogen, Carlsbad, CA).

### Statistical Analyses

SigmaStat (Systat Software Inc., San Jose, CA) software was used to compare groups by one- two- and three-way ANOVAs with Fisher LSD as the preferred post-hoc and to perform Pearson correlation coefficients. All groups were compared to their respective aCSF time point. Whenever the data from the aCSF groups were statistically identical this group is shown collapsed for simplicity. Graphs are shown with SEMs represented by error bars.

## Results

Overall, chronic infusion of LPS was well tolerated by all rats. Initially after surgery, all LPS-treated rats lost approximately 15% of body weight. Within two weeks, rats begin and continue to gain weight for the duration of the study.

### Behavior

**Figure 1:**
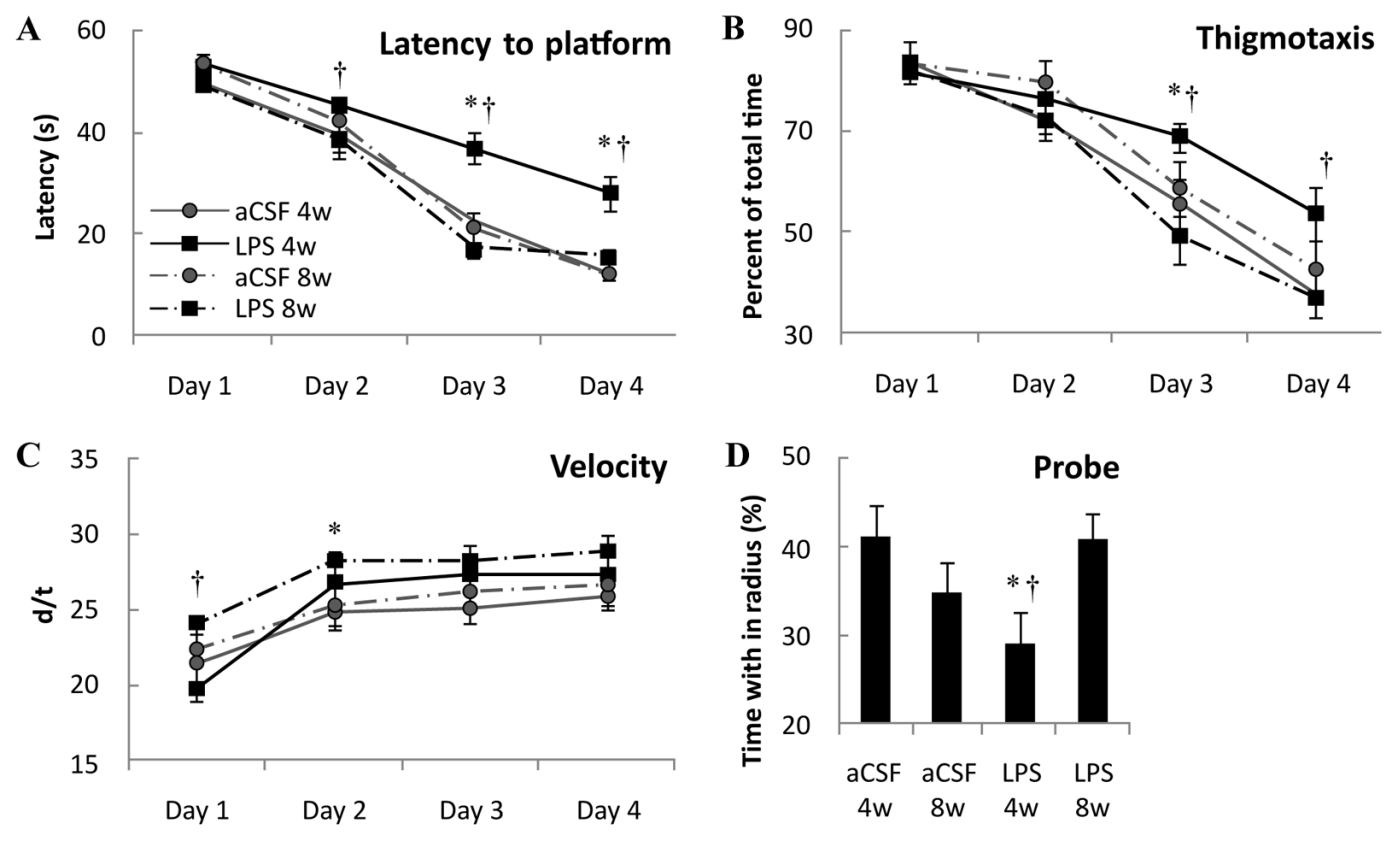
Morris water maze performance. Rats infused with LPS for 4 weeks have increased latency to find the hidden platform (A) compared to aCSF 4w on days 3 and 4 (*p*<0.001^*^) and LPS 8w on testing days 2 through 4 (*p* ≤ 0.041^†^). LPS 4w spent a greater percentage of time in the perimeter of the pool (B) than both aCSF 4w and LPS 8w (*p* ≤ 0.008^*†^) on days 3 and 4. LPS 4w did not swim slower (C) than aCSF controls, however, LPS 8w swam faster than LPS 4w on day 1) and aCSF 8w on day 2 (*p*<0.05^*†^). LPS 4w spent less time within the radius of the absent platform (D) than did aCSF 4w (*p*=0.012^*^) and LPS 8w (*p*=0.016^†^).

Spatial learning memory was assessed using the Morris water maze on animals infused with LPS or aCSF for 4 or 8 weeks. The data were analyzed by two-way ANOVA. Analysis of the latency to find the hidden platform (Figure 1A) revealed a main effect of trial day in which all groups improved across days (F_3, 255_=146.7, p<0.001). Each group improved across each day (p≤0.01) except LPS 8w which consistently performed well and did not improve between days 3 and 4. There was a main effect of experimental group (F_1,255_=18.0, p<0.001) and interaction between experimental group and testing day (F_9,255_=3.1, p<0.05) in which LPS 4w took longer to find the hidden platform than aCSF 4w on days 3 and 4 (^★^p<0.001), and longer than LPS 8w on testing days 2 through 4 (†p≤ 0.05), which was not statistically different from aCSF 8w (p>0.05). Overall, LPS infusion for 4 weeks impaired spatial learning memory, but 8 weeks of LPS infusion did not.

Thigmotaxis, defined as the percentage of trial time spent swimming within 10 cm of the pool wall, was analyzed (Figure 1B). There was a main effect for testing day (F_3,255_=93.9, p<0.001) where each group spent a significantly reduced percentage of time in the outer perimeter of the pool across days 2, 3 and 4 (p≤ 0.01), except for rats in the LPS 4w group who demonstrated reduced thigmotaxis between testing days 3 and 4 (p<0.001), but not between days 1, 2 and 3 (p>0.05). There was a main effect for experimental group (F_3,255_=6.4, p<0.001) and interaction between experimental group and testing day (F_9,255_=6.4, p<0.001) in which LPS 4w spent a greater percentage of trial time along the wall of the pool than both aCSF 4w and LPS 8w (p≤0.01). These data complement the latency analysis, showing that LPS 4w spent a greater percentage of trial time in the pool perimeter as an alternative to locating the platform indicating that they choose a poor strategy or did not understand the task.

Average swim speed (Figure 1C) was analyzed and there was a main effect of testing day on speed (F_3,255_=25.3, p< 0.001) whereby all groups increased swim speed between days 1 and 4 of testing (p ≤ 0.006) and a main effect for experimental group (F_3,255_=7.0, p<0.001) in which LPS 8w swam with greater speed than LPS 4w on day 1 (p< 0.001) and greater speed than aCSF 8w on day 2 (p<0.05). Swim speed of LPS 4w was not significantly different from aCSF 4w (p>0.05), therefore reduced velocity does not account for the increased latency to find the hidden platform. The probe trial (Figure 1D) revealed a main effect of experimental group (F_3,64_=6.82, p<0.05) such that LPS 4w spent less time within the radius of the absent platform than did aCSF 4w (p<0.01) or LPS 8w (p<0.05). These data, like latency to find the hidden platform, indicate that rats were impaired after 4 weeks of LPS infusion, but were no longer impaired after 8 weeks of LPS infusion.

Overall, these data indicate that chronic LPS infusion over 4 weeks impaired spatial memory performance, but that this impairment was no longer present after 8 weeks of LPS infusion.

### Histology

#### Hippocampus

**Figure 2:**
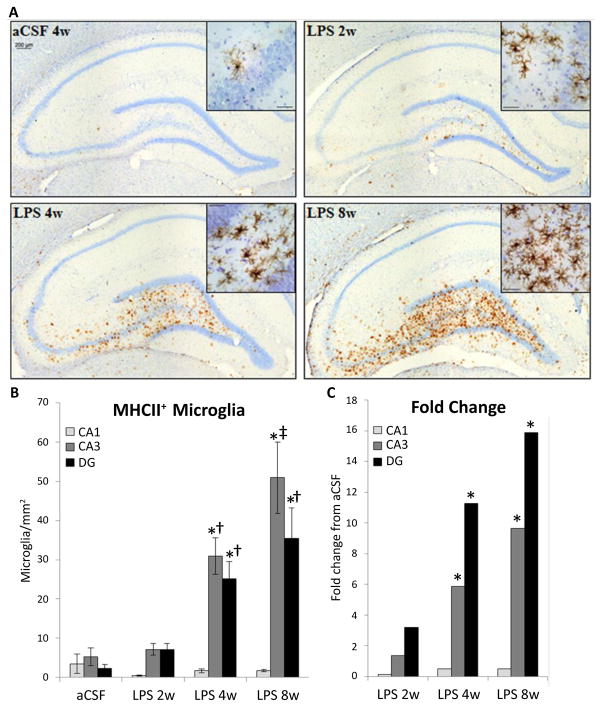
Distribution of MHC II-IR microglia (brown cells) within the hippocampus counterstained with cresyl violet (A) at 10X and 20X (insert). Scale bar = 200 μm. Number of MHC II-IR cells was counted per area (B). LPS infusion lead to an increased in the density of MHC II-IR microglia in hippocampal subregion; CA3>CA1 of LPS 2w (p=0.041) and CA3>DG>CA1 in LPS 4w and LPS 8w (p≤0.022). Within the DG and CA3, LPS 4w and LPS 8w had significantly more MHC II-IR microglia than aCSF controls and LPS 2w (p≤0.001^*†^). In the CA3, LPS 8w rats had significantly more MHC II-IR microglia than LPS 4w rats (p=0.029^‡^).

The distribution of activated microglia, defined by MHC II expression, is consistent with our previous reports that some brain regions have a greater proclivity than others to express MHC II-IR microglia in response to the continued presence of LPS [26,30]. MHC II-IR microglia were identified immunohistochemically (Figure 2A) and manually counted within the CA1, CA3 and DG sub-regions on four dorsal hippocampal slices per animal. A three-way ANOVA revealed main effects of inflammatory treatment (F_1,171_=37.09, p<0.001, Figure 2B, 2C), duration of treatment (F_2,171_=7.34, p<0.001) and hippocampal region (F_2,171_ = 12.91, p<0.001) as well as an interaction between duration and inflammatory treatment (F_2,171_=6.52, p=0.01) and an interaction between hippocampal region and inflammatory treatment (F_2, 171_=10.96, p<0.001). There are no significant differences in the number of MHC II-IR microglia between sub-regions in aCSF groups or LPS 2w (p>0.05). In contrast, LPS 4w and LPS 8w both express more MHC II-IR microglia in CA3 and DG than the CA1 sub-region (p<0.001), and LPS 8w have more MHC II-IR microglia in CA3 than DG (p=0.017). No groups are significantly different within the CA1 sub-region (p>0.05). Within the DG and CA3, LPS 4w and LPS 8w both express more MHC II-IR microglia than respective aCSF controls (p<0.001) and LPS 2w (p≤0.01). LPS 8w has more MHC II-IR microglia than LPS 4w within the CA3 sub-region (p<0.001).

While rats infused with LPS for 4 weeks performed poorly in the Morris water maze task, there is no correlation (p<0.05) between the number of MHC II-IR microglia in any of the hippocampal sub-regions investigated with latency to find the hidden platform or perseverance in the region of the missing platform during the probe trial, and this holds true for rats infused with LPS for 8 weeks and aCSF-infused animals as well.

**Figure 3:**
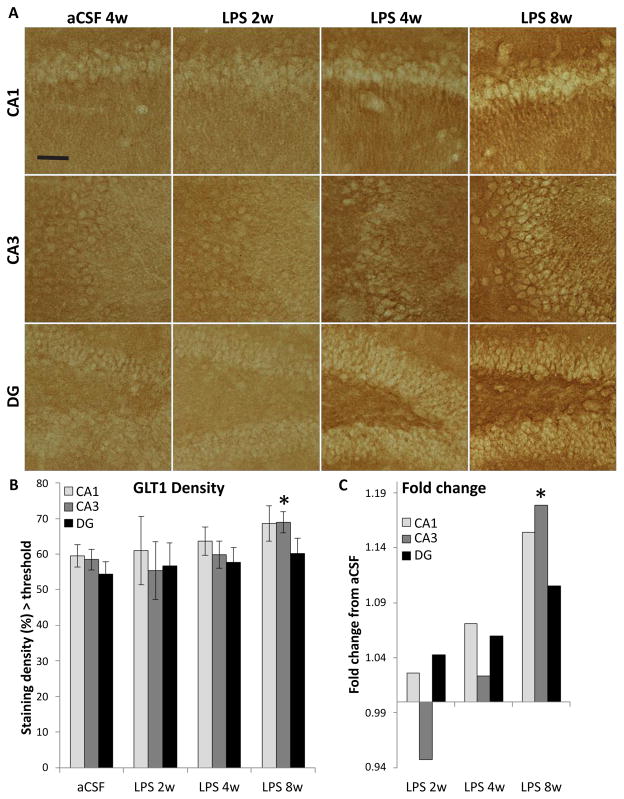
GLT1-IR within the CA3 of the hippocampus. Shown at 10 X (A), staining density was evaluated (B) and expressed in LPS-infused rats relative to controls (C). Scale bar=200 μm. The density of GLT1 showed a significant main effect of experimental group (*p*<0.001) and region (*p*<0.05), i.e. more GLT1 staining was observed in LPS 8w than aCSF controls (*p<0.01).

The density of GLT1glutamate transporter sites (Figure 3A) showed a significant main effect of experimental group (F_5,780_=4.9, p<0.001) and region (F_2,780_=3.4, p<0.05, Figure 3B). More GLT1 staining was observed in LPS 8w than their aCSF controls (p<0.01, Figure 3C). Overall, LPS exposure increased GLT1 density time-dependently. LPS 8w, which improved in the water maze compared to LPS 4w, had significantly more GLT1 in the CA3 than controls (p<0.05).

**Figure 4:**
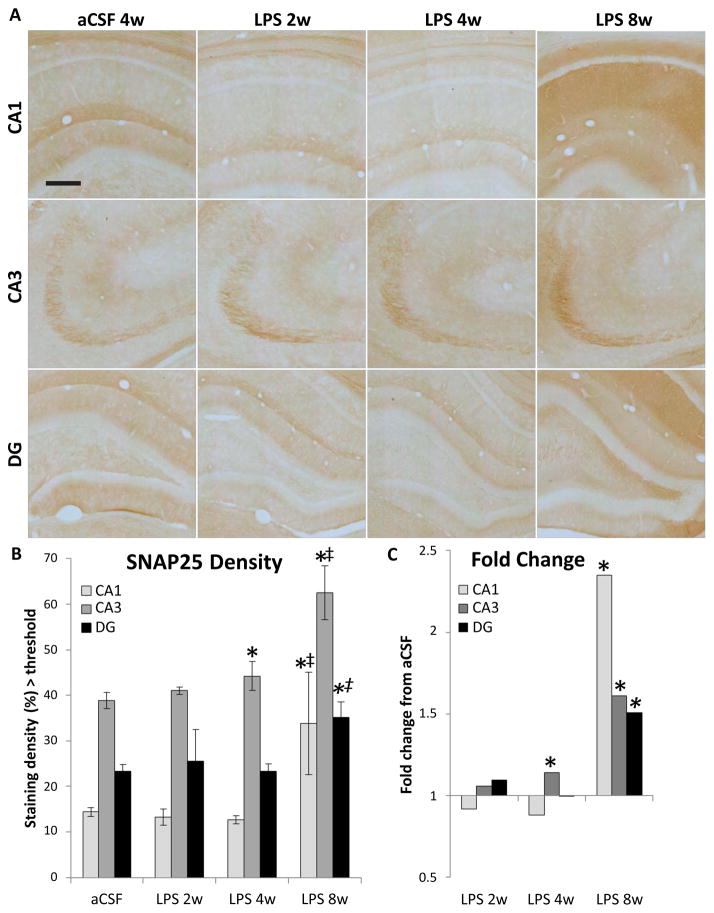
SNAP25-IR within the hippocampus. Shown at 10X (A), staining density was evaluated (B) and expressed in LPS-infused rats relative to controls (C). Scale bar = 800 μm. There was a higher density of staining in LPS 8w than aCSF 8w (p<0.001^*^) and compared to LPS 2w and LPS 4w (p≤0.015^†‡^). For all groups CA3>DG>CA1 (p≤0.014), except aCSF 2w in which CA3 and DG are not significantly different. SNAP25-IR increased in the CA3 region of LPS 4w as compared to aCSF controls (p=0.004^*^). LPS 8w expressed more SNAP25 in all regions than aCSF 8w (p≤0.046^*^), in CA1 and CA3 compared to LPS 4w (p≤0.031^†^) and compared to LPS 2w in CA1

SNAP25 is a synaptic docking protein, and levels of SNAP25 may reflect the docking and release of vesicles containing glutamate. SNAP25 was quantified as density of immunostaining above a determined threshold per area (Figure 4A). A three-way ANOVA revealed main effects of inflammation group (F_1,110_=14.56, p<0.001), duration of infusion (F_2,110_=14.34, p<0.001) and hippocampal region (F_2,110_=83.74, p<0.001) as well as an interaction between inflammation group and duration of infusion (F_2,110_=10.77, p<0.001, Figure 4B). SNAP25 staining is more dense in the CA3 of LPS 4w than aCSF 4w (p<0.01). LPS 8w had a greater staining density than aCSF 8w in all three subregions (p≤0.05), than both LPS 2w and LPS 4w in the CA1 and CA3 (p<0.001) and greater than LPS 4w in DG (p<0.01, Figure 4C).

Neither SNAP25 nor GLT1 correlated with WM performance on the final day of testing; however, SNAP25 in CA3 correlated with fewer entrances in the vicinity of the missing platform during the probe trial (r = −1, p<0.01) and GLT1 in CA3 correlated positively (r = 0.938, p<0.01) with more entrances.

#### Substantia Nigra and Locus Coeruleus

**Figure 5:**
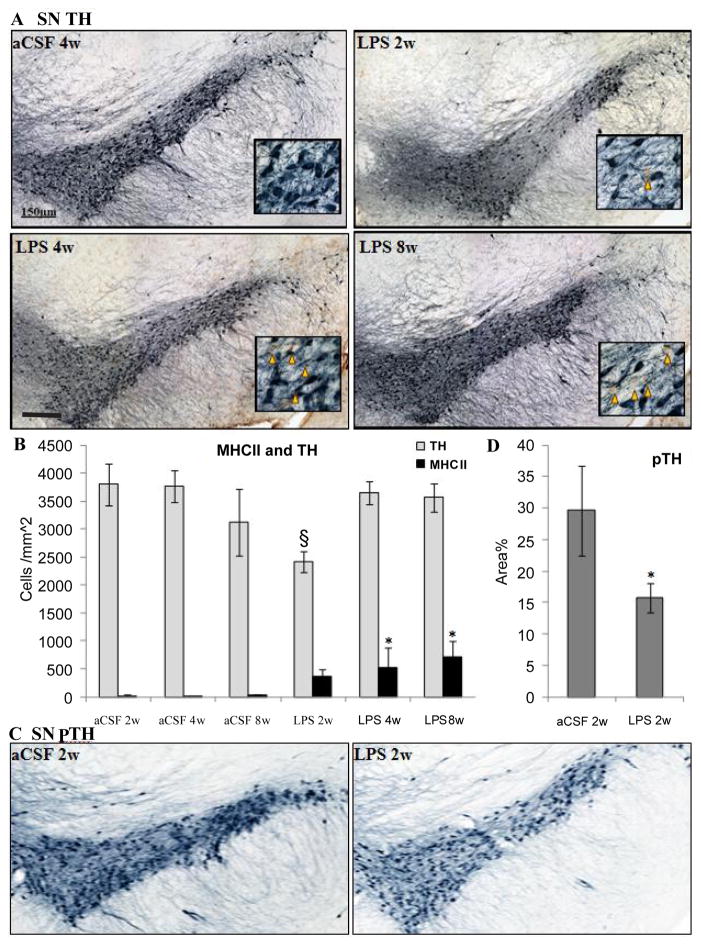
Distribution of TH-IR neurons (blue) and MHC II-IR microglia (brown). (A) The SNpc (A) at 10 X with inset at 40 X (orange triangles mark the site of MHC II-IR microglia). Scale bar=150 μm. Quantification of TH in the SNpc (B) demonstrates that there is a reduction in the number of TH-IR neurons after chronic LPS infusion for 2 weeks compared to aCSF controls (*p*=0.025^§^)., but not after 4 or 8 weeks LPS infusion. MHCII-IR microglia number is increased after 4 and 8 weeks LPS infusion compared to aCSF controls (p=0.031^*^). There is significantly less staining density of pTH in LPS 2w than aCSF 2w (C,D *p*<0.025).

Within the SNpc, a one-way ANOVA determined that LPS-infused rats had more MHC II-IR microglia than aCSF rats (F_1,54_=4.9, p<0.05) and the number of TH-IR cells was significantly (F_5,54_=2.9, p<0.05) reduced in LPS 2w rats as compared to all other groups (Figure 5A, 5B). Dopamine synthesis is controlled by the phosphorylation state of TH [33], and density of pTH-IR cells was also significantly (p<0.05) decreased by LPS two weeks after the initiation of the infusion (Figure 5C, 5D). Within the LC, there was a main effect between experimental groups (F_5,338_=14.14, p<0.001) demonstrating a decrease in TH staining after 2 weeks LPS infusion (p<0.001) that was resolved by 4 weeks (Figure 7A, 7B).

**Figure 6:**
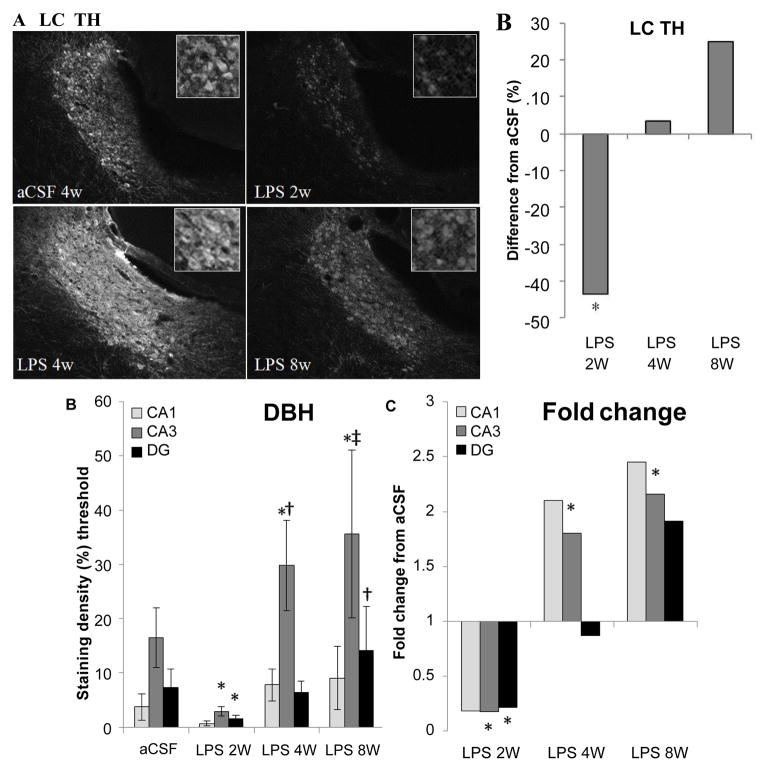
Integrity of the LC projection to the hippocampus. Within the LC, there is a decrease in TH staining (A) after 2 weeks LPS infusion (*p*<0.001) that is resolved by 4 weeks (B). Compared to controls, DBH staining intensity was reduced in CA3 and DG of LPS 2w and elevated in CA3 of LPS 4w and LPS 8w (*p≤ 0.007, C). DBH staining is more dense in LPS 4w CA3 and LPS 8w DG (†p≤ 0.028) than LPS 2w in the corresponding regions. LPS 8w CA3 is more dense with DBH staining than both LPS 2w and LPS 4w (‡p≤ 0.009, D).

Noradrenergic projections from the LC were evaluated by examining DBH in hippocampus. A two-way ANOVA determined that there were main effects of experimental group (F_5, 473_=12.71, p<0.001), hippocampal region (F_2,473_=35.93, p<0.001) and an interaction between the two (F_10,473_=3.32, p<0.001, Figure 6C, 6D). Within experimental groups, there is less DBH staining in LPS 2w than aCSF 2w (p < 0.001), while LPS 4w and LPS 8w show greater staining density than their aCSF counterparts (p<0.001) and LPS 8w > LPS 4w > LPS 2w (p≤0.05). LPS 2w has less DBH-IR than aCSF 2w in both CA3 and DG (p 0.01), while both LPS 4w and LPS 8w demonstrate more staining in CA3 than their aCSF controls (p<0.001). Within CA3, LPS 8w > LPS 4w> LPS 2w (p≤0.01) and LPS 8w has a higher density of staining in the DG than LPS 2w (p<0.05). DBH staining is more dense in CA3 that both DG and CA1 within aCSF 2w, LPS 4 and LPS 8w groups (p<0.001) and within aCSF 8w CA3 DBH staining is more intense than CA1 (p<0.05). Taken together, these data suggest that the LC projections to anterior hippocampus are effected by chronic neuroinflammation. Like TH in the brainstem and LC, DBH staining in the hippocampus correlated significantly (p<0.05) with an increase in MHC II-IR microglia.

### NO release from BV-2 microglia cell culture

**Figure 7:**
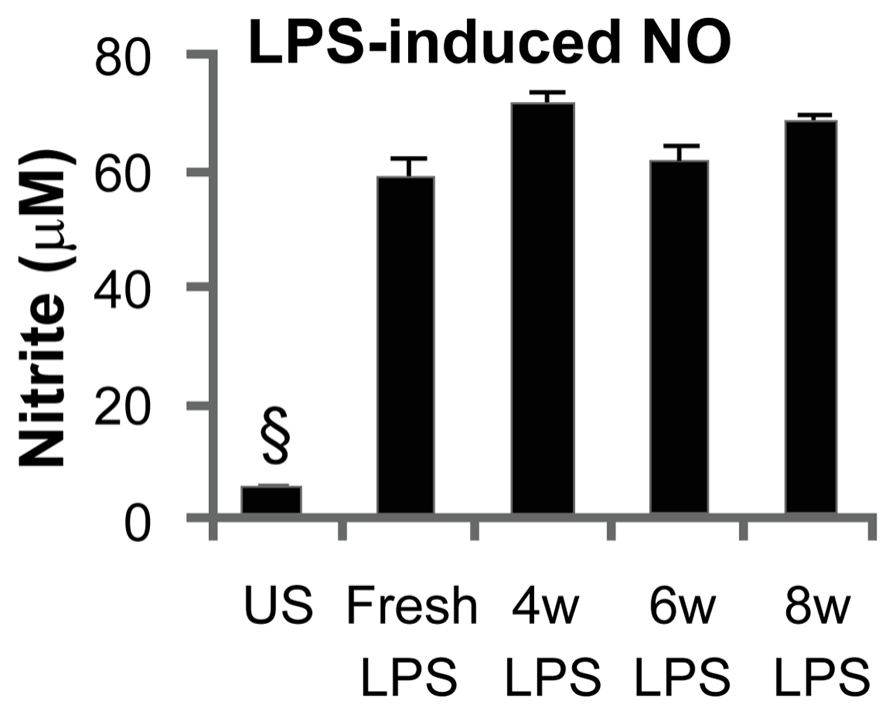
LPS maintains potency after incubation in osmotic minipump. Cultured BV-2 microglia cells responded to fresh LPS as well as LPS that had been incubated in an Alzet osmotic minipump for 4, 6 or 8 weeks (at 37° C in 0.9% saline bath) with the release of similarly increased levels of NO, as compared to un-stimulated cells (§p<0.003).

LPS stored in an osmotic mini-pump for 4, 6 or 8 weeks elicited a significantly (F_4,30_=161.2, p<0.001, by one-way ANOVA) elevated and equivalent release of NO from a BV-2 microglia cell line as freshly prepared LPS solution (Figure 7). These results confirmed the viability of LPS within the osmotic minipumps at body temperature.

## Discussion

The results document a series of compensatory processes related to the regulation of glutamate that developed in response to a chronic pro-inflammatory stimulus. In the current study, infusion of LPS into the IV^th^ ventricle induced microglial activation and MHC II expression in regions typically vulnerable to age-associated degenerative diseases such as the hippocampus, SN and LC, as well as impaired spatial memory and reduced TH expression in the SN and DBH expression in the hippocampus. The number of MHC II-IR microglia within the hippocampus and SN continued to increase as LPS-induced spatial memory deficit and reduced TH and DBH expression attenuated.

### Chronic activation of microglia

LPS directly activates toll-like receptor 4 (TLR4) on microglia, eliciting the expression of MHC II on microglia scattered throughout the brain within only a few days [21]. Increasing numbers of microglia expressed MHC II over the 8 week LPS infusion period, suggesting an expanding and progressive activation of microglia upon exposure to LPS over time; although LPS is lipid soluble and widely dispersed, our results suggest not all microglia are activated simultaneously. In addition, within the first few days the hippocampus showed very few MHC II microglia; the density here gradually increased as it declined in other brain regions [21]. This pattern of activation in the presence of LPS may recapitulate an important aspect of the inflammatory process in the diseased brain characterized by activation of individual microglia and groups of microglia within specific brain regions over time due to the accumulation of aberrant protein, such as β-amyloid or α-synuclein, and presence of degenerating neurons in the microenvironment.

Activation of microglia by LPS is sufficient to drive spatial memory impairment and decline in TH expression that are not sustained by continued LPS infusion. This apparent disconnect between increased microglia MHC II expression and both attenuated deficits in water maze performance and TH expression is consistent with the hypothesis that MHC II is residually expressed on microglia that have converted from an ‘active’ pro-inflammatory state, or classical M1, into an alternative activation state, called M2a [34]. Simply stated, MHC II is not an accurate indicator of the pro-inflammatory status of the resident microglia, thus its presence did not correlate with performance or changes in TH expression. Microglia chronically exposed to LPS for up to 8 weeks in our model will undoubtedly develop endotoxin tolerance [35] and may residually express MHC II, but no longer maintain a pro-inflammatory profile. Thus, the presence of MHC II-IR microglia over time may only represent the accumulation of microglia that were initially activated by the LPS exposure but are no longer in an active pro-inflammatory state as well as newly activated populations of microglia. Endotoxin tolerance itself may be an important compensatory mechanism to protect the brain from the detrimental consequences of prolonged neuroinflammation [36]. We hypothesize that as additional microglia become activated at different time points throughout the 8 week infusion period, so too do microglia gradually become tolerant throughout that period. Therefore, a chronic neuroinflammatory environment is established not by microglia that remain active, but by the sequential activation of additional microglia over time. We speculate that the young brain compensates to a pro-inflammatory challenge via a complex regulation of glutamatergic neurotransmission that protects neurons from injury; this compensatory process may become impaired during the normal or pathological aging subsequently predisposing the brain to neurodegenerative disease.

### Time-dependent attenuation of response to LPS in SN and LC

Continuous LPS infusion for up to eight weeks was characterized by an accumulation of microglia expressing MHC II within the DG and CA3 regions of the hippocampus and a significant impairment in hippocampal-sensitive spatial working memory after four weeks of infusion that was completely attenuated eight weeks after the initiation of the infusion. Likewise, infusion of LPS into the IV^th^ ventricle increased the number of activated microglia within the SN and throughout the brainstem. The number of dopaminergic neurons in the SN expressing TH and pTH were reduced after 2 weeks of LPS infusion, but restored after 4 weeks. Similarly, the number of noradrenergic neurons in the LC expressing TH and the density of noradrenergic projections to the hippocampus distinguished by DBH expression were reduced after 2 weeks of LPS infusion and restored 4 weeks later. Decreased availability of the rate-limiting enzymes TH and DBH suggests reduced production of dopamine and norepinephrine. Diminished production of neurotransmitters or, ‘luxury systems,’ may be a cellular defense strategy to divert energetic resources to survival mechanisms in a chronically stressful environment. Interestingly, reductions in TH and DBH driven by the immune response to LPS are not sustained over 4 and 8 weeks, though the inflammatory environment persisted or was elevated in the SN, LC and hippocampus. These data also suggest that the low-level chronic neuroinflammation produced by chronic infusion of LPS does not produce neuronal cell death; consistent with lack of observed neurodegeneration in previous studies of the hippocampus and temporal lobe region [22]. Taken together, these results suggest that the young brain compensates on a behavioral and cellular level to the chronic presence of neuroinflammation and continued activation of microglia.

### Compensation within the Hippocampus through Regulation of Glutamate

Within the hippocampus our results are consistent with the hypothesis that compensatory changes related to synaptic regulation of glutamate might underlie the recovery of spatial working memory in the continued presence of LPS and increased number of MHC II-IR microglia; therefore, we choose to pursue this mechanism further. We have previously documented the impact of glutamatergic dysregulation within the hippocampus following four weeks of LPS infusion. Rats demonstrated impaired accuracy of neural encoding, information processing and spatial memory that was restored by pharmacologically antagonizing glutamatergic-NMDA receptors with the partial uncompetitive antagonist memantine [30,37,38]. Furthermore, a reduction of glutamate release by stimulation of endocannabinoid receptors or blockade of adenosine receptors by caffeine administration prevent LPS-induced microglia activation and spatial memory deficits [29, 39]. The prevention of LPS-induced microglia activation and behavioral deficit by pharmacological reduction of glutamate activity suggests that neuroinflammation leads to aberrant glutamate activity. These pharmacological observations are complemented by evidence from the current study that naturally occurring changes in glutamate regulation, i.e. increased expression of SNAP25 and GLT1, occur as performance in the water maze task recovers.

SNAP25 is part of a large protein complex involved in release of neurotransmitter from vesicles and learning and memory. Decreased levels of SNAP25 expression have been described in disease states characterized by increased network excitability and possibly associated with neurotoxicity [40] and in elderly individuals with Alzheimer’s and Down syndrome who also Alzheimer’s like pathology and cognitive impairment [41]. SNAP25 did not decrease upon exposure to the neuroinflammatory environment created by chronic LPS infusion; however SNAP25 expression did progressively increase between 4 and 8 weeks of infusion, concurrent with the attenuation of behavioral impairment. One interpretation is that, during a time of recovery, increased SNAP25 in the glutamatergic terminals that abundantly populate the hippocampus may indicate decreased glutamate release; a property which may be beneficial against a background of inflammation-induced elevation of extracellular glutamate and is consistent with the beneficial profile of memantine which blocks activation of NMDARs by excessive extracellular glutamate but not synaptically evoked glutamate release [42]. Conversely, SNAP25 negatively regulates native voltagegated calcium channels in glutamatergic neurons at glutamatergic terminals [43], suggesting that increased SNAP25 above basal levels may lead to decreased depolarization and neurotransmitter release from the pre-synaptic terminal. Therefore the increased expression of SNAP25 in the current study may indicate decreased glutamate exocytosis from glutamatergic neurons, a restoration of hippocampal sparse encoding and recovered spatial memory similar to what we have previously described following administration of glutamatergic receptor channel antagonists [37].

An increase in extracellular glutamate may result from inflammation-induced alterations in the ability of astrocytes to sequester glutamate [44]. Astrocytes exhibit only partial endotoxin tolerance [36], and effects of LPS upon glutamate uptake may be exacerbated by continued LPS infusion. GLT1 is the principle excitatory amino acid transporter responsible for approximately 80–90% of glutamate clearance from the extracellular space in hippocampal tissue [45, 46]. Reduction of glutamate transport and clearance as well as variants of the GLT1 gene have been associated with aging and AD [47–49]. If reduced transport of glutamate by astrocytes in fact contributes to elevated glutamate levels, this may be due to a change in transporter activity, as we did not observe a decrease in GLT1 expression. However, increased expression of GLT1 was observed in the CA3 and DG of the hippocampus after 4 and 8 weeks of LPS infusion, and indicates that glutamate clearance may be enhanced. Increased glutamate clearance may compensate against the effects of chronic neuroinflammation and improve spatial memory by reducing synaptic ‘noise’ from elevated extracellular glutamate. This interpretation is consistent with compensatory increase in glutamate clearance rate seen in aged rats after elevated evoked-glutamate release in middle age [50], and a similar compensation seen in a genetic model of consistent-low level lifetime elevated glutamate release [51, 52].

Compensatory increases in the SNAP25 protein that regulates glutamate release and GLT1 which sequesters glutamate into astrocytes are consistent with a previous finding that NMDARs after 4 weeks of LPS infusion [22]; another compensatory change that would act to decrease responsiveness to elevated extracellular glutamate. Taken together, these data indicate that naturally occurring changes within the hippocampus that decrease the action of glutamate on the post-synaptic site and occur during the continued activation of microglia, represent a process of compensation against chronic neuroinflammation that proceeds and persists throughout behavioral recovery.

Our results are consistent with the hypothesis that compensatory changes related to synaptic regulation of glutamate might underlie the recovery of spatial working memory in the continued presence of LPS and increased number of MHC II-IR microglia. The unique mechanisms discovered in this study by which the young brain compensates against chronic exposure to an inflammatory environment, namely an increase of GLT1, shed light on the interaction between neuroinflammation, glutamatergic regulation and compensatory mechanisms that may prolong the period of health before the manifestation of pathology and clinical symptoms in neurodegenerative disease. Taken together, these data indicate that naturally occurring changes that decrease the action of glutamate within the hippocampus of the young adult brain in an environment of persistent, low-level neuroinflammation represent a process of compensation that proceeds and persists throughout behavioral recovery in the young brain. These observations are consistent with the hypothesis that compensatory biochemical processes within the young brain are able to delay the clinical symptoms of degenerative diseases; with advanced age these processes may fail to provide the necessary compensation of function or respond positively to anti-inflammatory therapy [53]. Therefore, if we can pharmacologically drive an increase in glutamate clearance, we may be able to facilitate a compensatory response that would be sufficient to attenuate inflammation-induced cognitive impairment and potentially be efficacious against the manifestation of clinical symptoms of AD.

## Abbreviations

aCSF: Artificial cerebral spinal fluid
AD: Alzheimer’s Disease
DG: Dentate Gyrus
GDNF: Glial cell line-derived neurotrophic factor
GLT1: Glutamate Transporter Type 1 (excitatory amino acid transporter type 2)
i.p: Intraperitoneal
IL-1β: Interleukin-1-Beta
LC: Locus Coeruleus
LPS: Lipopolysaccharide
MHC II: Major Histocompatibility Complex II
NO: Nitric Oxide
PD: Parkinson’s Disease
pTH: Phosphorylated TH
PGE2: Prostaglandin E2
SN: Substantia Nigra
SNpc: Substantia Nigra Pars Compacta
SNAP25: Synaptosomal-associated Protein −25 kd
TH: Tyrosine Hydroxylase
TLR: Toll-Like Receptor
TNFα: Tumor Necrosis Factor-α
